# Evidence for Cross-Tolerance to Nutrient Deficiency in Three Disjunct Populations of *Arabidopsis lyrata* ssp. *lyrata* in Response to Substrate Calcium to Magnesium Ratio

**DOI:** 10.1371/journal.pone.0063117

**Published:** 2013-05-01

**Authors:** Maren E. Veatch-Blohm, Bernadette M. Roche, MaryJean Campbell

**Affiliations:** Biology Department, Loyola University Maryland, Baltimore, Maryland, United States of America; United States Department of Agriculture, Agricultural Research Service, United States of America

## Abstract

Species with widespread distributions that grow in varied habitats may consist of ecotypes adapted to a particular habitat, or may exhibit cross-tolerance that enables them to exploit a variety of habitats. Populations of *Arabidopsis lyrata* ssp. *lyrata* (L.) O’Kane & Al-Shehbaz grow in a wide variety of edaphic settings including serpentine soil, limestone sand, and alluvial flood plains. While all three of these environments share some stressors, a crucial difference among these environments is soil calcium to magnesium ratio, which ranges from 25∶1 in the limestone sand to 0.2∶1 in serpentine soil. The three populations found on these substrates were subjected to three different Ca to Mg ratios under controlled environmental conditions during germination and rosette growth. Response to Ca to Mg ratio was evaluated through germination success and radicle growth rate, rosette growth rate, and the content of Ca and Mg in the rosette. All three populations were particularly efficient in fueling growth under nutrient deficiency, with the highest nutrient efficiency ratio for Ca under Ca deficiency and for Mg under Mg deficiency. Although the serpentine population had significantly higher leaf Ca to Mg ratio than the limestone or flood plain populations under all three Ca to Mg ratios, this increase did not result in any advantage in growth or appearance of the serpentine plants, during early life stages before the onset of flowering, even in the high Mg substrate. The three populations showed no population by substrate interaction for any of the parameters measured indicating that these populations may have cross-tolerance to substrate Ca to Mg ratio.

## Introduction

Highly stressful environments, such as serpentine barrens, are associated with a high level of endemism [1]–[5], but are also host to species with widespread distribution in environments ranging from very moderate to extreme stress [6]–[9]. These widespread species, known as bodenvags when they occur on serpentine and nonserpentine soils, may flourish due to specialization or due to a more generalized response. Specialized ecotypes may result from local adaptation. Alternatively, adaptive phenotypic plasticity may also play a role in enabling the plant to respond to environmental heterogeneity with a generalist strategy where responses to different stressors are due to developmental plasticity [10]–[14]. However, these are not the only two alternative states: Pigliucci [14] provides a useful framework for discussing the role of plasticity in specialists and generalists. Specialized, locally adapted ecotypes may exhibit plasticity, but this plasticity would be neutral or maladaptive (they do worse in non-native environments) [10]. Alternatively, generalists may not exhibit plasticity but instead may exhibit an average genotype that works adequately across all environments (“Jack-of-all-trades-master-of-none”). This type of generalist may exhibit cross-tolerance to different stressors. Cross-tolerance would be implicated if populations flourish in alien environmental conditions..

Calcium (Ca) to magnesium (Mg) ratio varies greatly in different soil types [7], [15]–[17], and this ratio can have a drastic effect on plant growth [18]–[19]. Plants have a wide range of strategies to handle variations in soil Ca and Mg. In some cases the Ca and Mg uptake by plants reflects the nutrient composition of their parent soils [5], [19]–[20]. Some plants grow well in all environments, regardless of the Ca to Mg ratio, such as serpentine and non-serpentine races of the moss *Funaria flavicans* [21]–[22]. In contrast, species may require high levels of their soil’s predominant cation while having little or a negative response to the less abundant cation. For example, *Poa curtifolia*, from serpentine soils, has a requirement for high Mg and has little response to increased Ca [1] and *Helianthemum chamaecistus,* from calcareous soils, has very low tolerance to even moderate levels of Mg [18]. Some species appear to have enzymatic adaptations that are related to high Mg content, such as serpentine races of *Festuca rubra* that have phosphatases on the root surface that have higher activity when exposed to high Mg concentrations [23]. In addition, some plants may have as yet unknown tolerance mechanisms that counteract the toxic effects of high Mg without the need for Mg exclusion [24]. Other species have increased or more efficient Ca uptake [2]–[3], [7], [9], [15], [22], [25]. Due to this diverse array of responses to Ca to Mg ratio, it is difficult to make generalizations about plant adaptations to variations in soil Ca and Mg content [26].

*Arabidopsis lyrata* ssp. *lyrata* (L.) O’Kane & Al-Shehbaz (Brassicaceae) is a mostly self-incompatible biennial/perennial with a widespread but fragmented distribution [27]–[32]. For example, in the North Eastern and Mid-Atlantic region of the United States *A*. *l*. ssp. *lyrata* is found growing in rock, shale, calcareous sand, serpentine soil, and sandy loam of alluvial flood plains. These environments present a series of both common and unique stressors. Most of these environments share the feature of being low competition environments, which is not unusual for plants that have such disjunct distributions [6], [33]–[34]. In almost all of these edaphic settings, *A*. *l*. ssp. *lyrata* experiences a period of low water availability [11], [35]–[36]. In addition to drought, in the sand, rock, and shale habitats plants may frequently encounter low humus levels, with a concurrent low availability of essential plant nutrients [11], [16], [37]–[38]. Serpentine soils combine these traits with high levels of Mg and heavy metals such as nickel and chromium, all of which are toxic to most plants [35]. Serpentine soils are particularly deficient in Ca, phosphorus, and potassium [35], [39]–[40]. In order to grow in such a diverse array of environments, disjunct populations of *A*. *l*. ssp. *lyrata* may have different ecotypes due to genetic differentiation that suit them to specific stressors. While local adaptation in *A*. *l*. ssp. *lyrata* has not been conclusively demonstrated, a recent study of microsatellite markers revealed genetic differentiation between serpentine and calcareous populations [41]. Turner *et al*. [42] found evidence of population differentiation in polymorphisms of the *cax7* locus (Ca: Na antiporter), with granitic populations exhibiting high nucleotide diversity, and serpentine populations exhibiting very low nucleotide diversity at this locus. The purpose of this study was to determine if the ability of *A*. *l*. ssp. *lyrata* to grow in disjunct environments with a wide range of substrate Ca to Mg ratios shows evidence of physiological differentiation within *A*. *l*. ssp. *lyrata* populations or evidence of a more generalist strategy.

## Materials and Methods

### Ethics Statement

All necessary permits were obtained for the described field studies. Permission for collection at Perry Preserve was obtained from Matt Levy of the Nature Conservancy. Permission for collection at Pilot Serpentine Barren was obtained from Deborah Barber of the Nature Conservancy. Permission for collection at Jug Bay Wetlands Sanctuary was obtained from Chris Swarth Sanctuary Director.

### Seed Source

Seeds of *A. l.* ssp. *lyrata* were collected by maternal plant from three populations in June 2007 and June 2008. For all experiments, seeds were taken at random from 20 maternal half sib families. In addition to collecting seeds at each site, soil from each site was collected within one meter of plants, by removing the first 10 centimeters of loose substrate. Samples from four bags of soil were mixed and ground before being analyzed for Ca and Mg content using flame atomic absorption spectrometry (FAAS). Each soil was prepared for FAAS via wet digestion as described in Grusak [43] and Pomper and Grusak [44]. The extractable Ca and Mg content was determined by extracting the soil sample in 1 M Ammonium acetate (shaken for 24 h) and then analyzed via FAAS [45]. Soil pH was determined by mixing soil and distilled water in a 1 to 1 ratio [45]. The first group of seeds (Dover) was collected from plants growing on the Perry Preserve in the Dover Plains region of New York (41.4351° N, 73.3345° W), on calcareous limestone sand with high levels of Ca and low levels of Mg [46]–[47]. The second group of seeds (Pilot) was collected from a serpentine soil from the Pilot Serpentine Barrens of Maryland and Pennsylvania (39.4214° N, 76.1123° W), which is characterized by high levels of Mg and low levels of Ca and the presence of heavy metals such as nickel and chromium [2]–[3]. The third group of seeds (Jug Bay) was collected from the Jug Bay Wetlands Sanctuary population [48] (38.7847° N, 76.7017° W), which grows on a sandy loam soil without extreme Ca or Mg concentrations. Twenty seeds from each of 20 half-sib families were pooled for each experiment.

### Germination and Seedling Growth

Seed germination percent, germination rate, and seedling root growth in response to substrate Ca to Mg ratio were evaluated twice in Fall 2008. In each experiment seeds of *A. l.* ssp. *lyrata* populations from Dover, Pilot, and Jug Bay were surface sterilized for three minutes in a mixture of 50% ethanol and 0.1% Tween. The seeds were rinsed with 95% ethanol and dried. Seeds were then stratified in a 0.1% agar/Tween mixture at 4°C for 4 days in the dark. Following stratification the seeds were transferred, using sterile pipettes, to 2% agar plates containing one of the three Ca to Mg ratios listed: 1∶0.1 (4000∶400 µmol), 1∶1 (4000∶4000 µmol), and 0.1∶1 (400∶4000 µmol). The experiment was set up as a nested design, with plate nested within Ca to Mg ratio. For each run of the experiment, treatment was replicated six times and four seeds from each population were sown on each plate, for a total of 24 seeds from each population per treatment. Population placement within each plate was randomized, and plate position within the growth chamber was also completely randomized. Plates were placed vertically into a growth chamber at 20°C with a 14∶10 h day/night cycle.

The plates were in the growth chamber for 17 days. Plates were examined for germination every day. A seedling was considered germinated at visible radicle emergence. Germination rate was calculated using the following formula from Maguire [49]: GR = (G_1_/D_1_+ G_2_/D_2_+ …+ G_f_/D_f_), where G_1_ to G_f_ are the number of seeds germinated on the first through final day of counting (D_1_ to D_f_). The length of the radicle after emergence was also measured for five days starting with the day of germination. A picture was taken of the radicle under a dissecting microscope and root lengths were measured using Image J [50].

The slope of root growth regressed on day was used to compare the differential success of early seedlings from the three populations on each of the three Ca to Mg ratios. Germination percentage, germination rate, and seedling root growth rate were analyzed using the general linear model with run, population, Ca to Mg ratio, population x Ca to Mg ratio, and run x Ca to Mg ratio as factors in the model in JMP 8 [51]. For analysis plate was nested within Ca to Mg ratio.

### Rosette Growth

Due to difficulty in maintaining reasonable temperatures in the greenhouse during the summer months, we focused our experiment on rosette growth during the juvenile stage, before the onset of flowering. Juvenile growth in response to substrate Ca to Mg ratio, was analyzed three times (Fall 2007, Spring 2008, and Fall 2008). During the first run only plants from Dover (limestone sand) and Pilot (serpentine) were used. However, Dover and Pilot are both from locations where there is a high concentration of divalent cations. It was thought that there might be similar stress response to these cations. Therefore, plants from the Jug Bay population (alluvial sandy loam) were included in subsequent runs as these plants come from an environment with much more moderate and equal total levels of each ion (Table 1). Seeds of the *A. l*. ssp. *lyrata* populations Dover, Pilot, and Jug Bay were sterilized as described above. The seeds were planted in pre-wet Rockwool plugs and stratified at 4°C for 4 days. The seeds were placed in a growth chamber with a 14∶10 h day/night cycle at a temperature of 20°C, for 24 days. The plugs were soaked in a modified Hoagland’s Solution [52] every three days. The seedlings were transferred into larger Rockwool cubes on the day that treatment with the nutrient solutions began. The experiment was set up as a randomized complete block with eight blocks. Plants used in the experiment were chosen randomly from the larger tray, and the initial leaf number recorded. The seedlings from Dover had an average of 6.3±0.14 leaves, from Pilot had an average of 5.2±0.22 leaves, and from Jug Bay had an average of 7.6±0.25 leaves at the initiation of treatment.

**Table 1:** Soil characteristics for Dover, Pilot and Jug Bay populations of *A. l.* ssp. *lyrata*.

	Dover	Pilot	Jug Bay
Substrate	Limestone Sand	Serpentine	Sandy Loam
pH	7.7	7.7	4.8
Total Ca (µg·g^−1^)	635242	6987	2756
Total Mg (µg·g^−1^)	24707	40276	1454
Extractable Ca (µg·g^−1^)	206	13	71
Extractable Mg (µg·g^−1^)	91	39	20
Total Ca to Mg Ratio	25.7∶1	0.17∶1	1.9∶1
Extractable Ca to Mg Ratio	2.3∶1	0.3∶1	3.5∶1

The plants received approximately 100 mL of treatment solution every three days via drip irrigation. The nutrient solutions were modified from the modified Hoagland’s solution of Epstein and Bloom [52] to alter the ratio of Ca to Mg as described above for germination. Plant growth was measured weekly for six weeks. Plant growth was evaluated by counting the number of leaves in each rosette and by measuring the average diameter of the rosette (determined by taking two perpendicular diameter measurements per plant).

After six weeks the plants were harvested at the soil line and analyzed for relative water content, chlorophyll *a* and *b* content, and fresh weight. Relative water content was determined for a single leaf from each plant in four blocks as described by Bogeat-Triboulot *et al*. [53]. Chlorophyll content was determined on a single leaf from each plant in the four blocks not used for relative water content determination. Leaves from the same whorl of each rosette were chosen for chlorophyll analysis. Each leaf was homogenized in 5 mL of 80% acetone, with an additional 20 mL of acetone added before centrifugation for a final volume of 25 mL. The samples were centrifuged for 10 min at 2500 rpm at 10°C. The samples were analyzed with a light spectrophotometer at wavelengths of 663 nm and 646 nm [54]–[55]. Total chlorophyll content (*a*+*b*) was used for analysis.

The remaining plant biomass was dried at 80°C for 48 hours and then dry weight was recorded. Leaves of the dried plants were analyzed by FAAS to determine the Ca and Mg concentration within the rosette leaves [56]. A subsample, from a mixture of leaves from the whole rosette, was wet digested as described above for soil analysis. Nutrient efficiency ratio (NER) for both Ca and Mg were calculated according to Baligar *et al.* [57], on a per sample basis and then averaged.

Data was analyzed with the general linear model using the fit model platform of JMP 8 [51], with initial leaf number as a covariate, and run, block, population, Ca to Mg ratio, population x Ca to Mg ratio, and run x Ca to Mg ratio as factors in the model. The following transformations were done on the data to meet the assumption of normality for data analysis. Dry weight and relative water content were square root transformed. Total chlorophyll content was natural log transformed. Rosette Ca, Mg, Ca to Mg ratio and Ca and Mg NER were natural log transformed. Multiple comparisons were done using Tukey’s HSD. A *P*-value of ≤0.05 was considered significant throughout.

## Results

### Soil Analysis

There were large differences in total Ca to Mg ratio among the substrates of the three populations. Dover (limestone sand) had the highest total soil Ca to Mg ratio, which was much higher than the 1∶0.1 ratio used in the experiments, while Pilot (serpentine) had the lowest total soil Ca to Mg ratio, most similar to the 0.1∶1 ratio, and Jug Bay (alluvial sandy loam) had a moderate total soil Ca to Mg ratio, most similar to the 1∶1 ratio (Table 1).

### Germination and Seedling Growth

**Figure 1:**
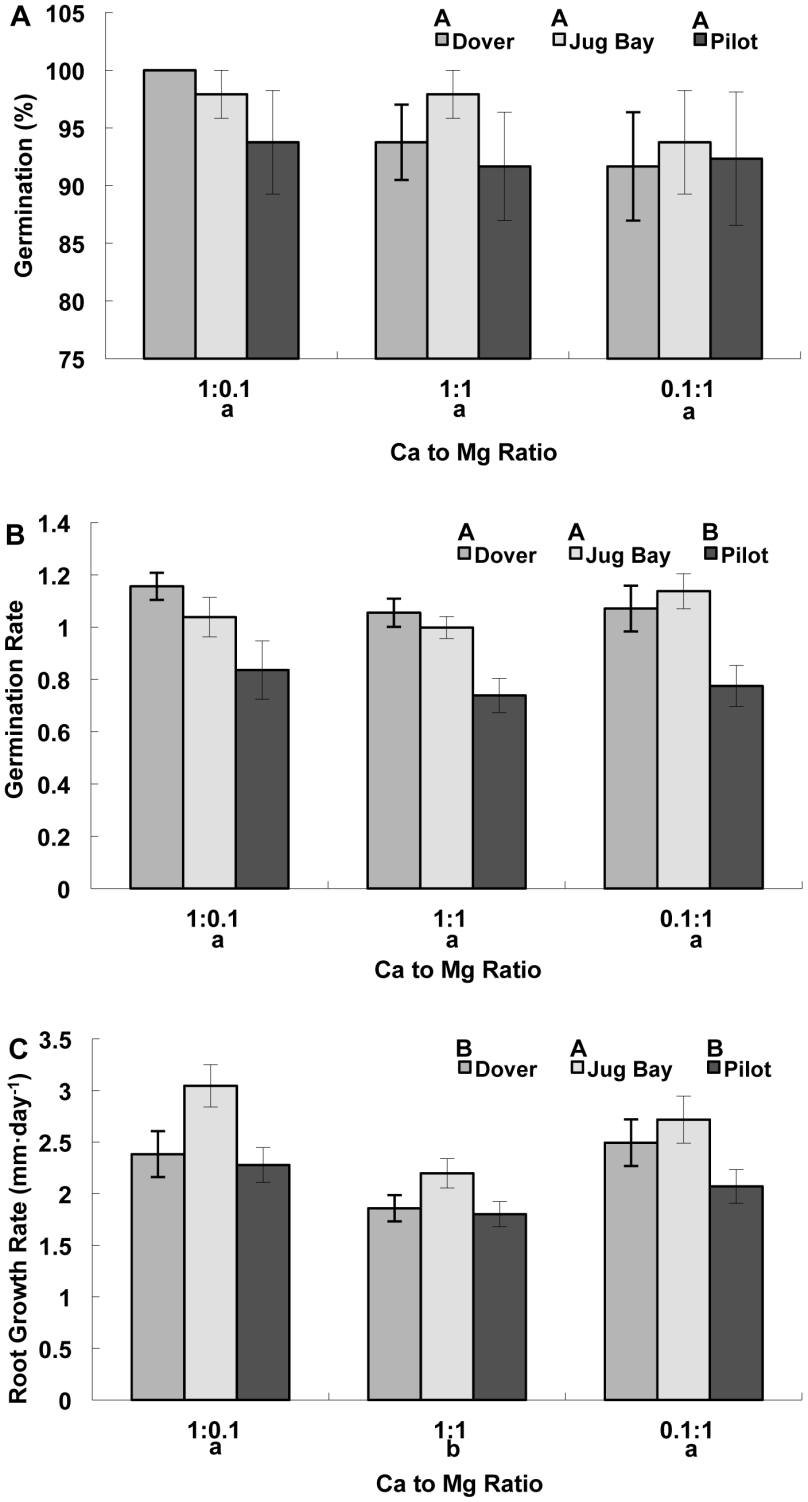
Seed germination and seedling root growth of three *A.l.* ssp. *lyrata* populations. **A.** Germination percentage, **B.** Germination rate, and **C.** Seedling root growth rate. All data are presented as a mean ± standard error (n = 12). The seeds were from serpentine (Pilot), limestone sand (Dover), and sandy loam (Jug Bay) soils. Different upper case letters indicate significant differences among populations, and different lower case letters indicate significant differences among substrate Ca to Mg ratios as separated by Tukey’s HSD (P<0.05).

Germination percentage was not significantly affected by population or by Ca to Mg ratio (Table 2, Fig. 1a). Calcium to Mg ratio did not have an effect on germination rate and root growth rate, but germination rate and root growth rate were significantly affected by population (Table 2). Pilot (serpentine) had a significantly lower germination rate than Dover (limestone sand) and Jug Bay (alluvial sandy loam) (Fig. 1 b). Early radicle growth rate was significantly higher in plants from Jug Bay than those from Dover and Pilot (Fig. 1c).

**Table 2:** ANOVA table for seed germination (percent and rate) and seedling root growth rate of three *A. l.* ssp. *lyrata* populations exposed to different substrate Ca to Mg ratios.

	Percent Germination			Germination Rate			Root Growth Rate		
*Source of Variation*	*df*	F	*P*	*df*	F	*P*	*df*	F	*P*
Run	1	0.01	0.9063	1	0.06	0.7797	1	73.89	<.0001
Plate Nested in Trt	5	0.53	0.7492	5	1.13	0.3500	15	2.42	0.0023
Population	2	1.02	0.3645	2	16.75	**<.0001**	2	8.93	**0.0002**
Ca to Mg Ratio	2	0.75	0.4754	2	1.02	0.3631	2	7.98	**0.0004**
Population x Ca to Mg Ratio	4	0.29	0.8820	4	0.50	0.7364	4	0.45	0.7705
Run x Ca to Mg Ratio	2	0.25	0.7775	2	2.71	0.0720	2	22.36	<.0001
Error	91			91			361		

### Rosette Growth

**Figure 2:**
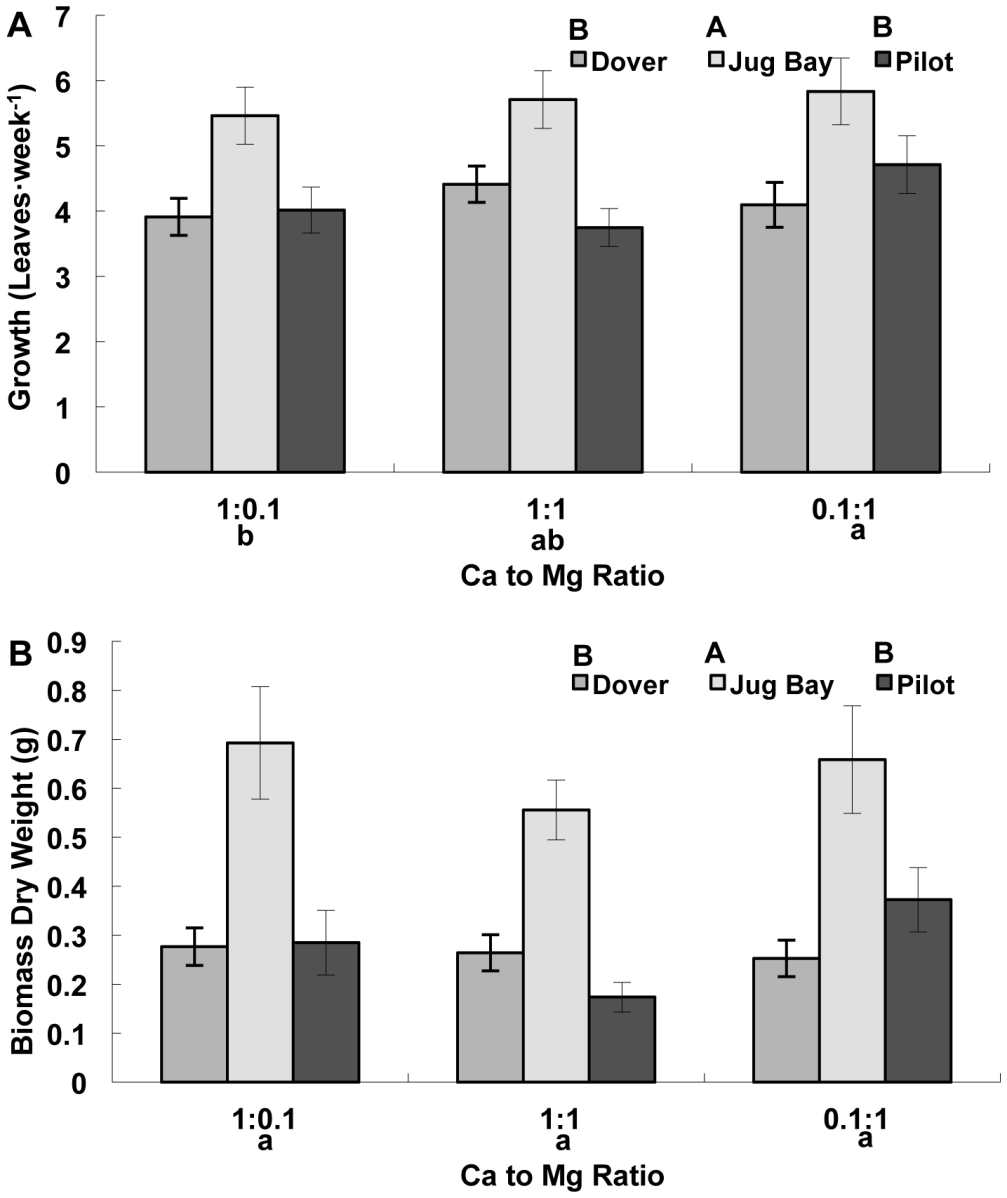
Rosette growth and biomass production of juvenile plants of three *A.l.* ssp. *lyrata* populations. **A.** Growth rate (leaves per week) and **B.** biomass (dry weight). All data are presented as a mean ± standard error (n = 16). The plants were from serpentine (Pilot), limestone sand (Dover), and sandy loam (Jug Bay) soils. Different upper case letters indicate significant differences among populations, and different lower case letters indicate significant differences among substrate Ca to Mg ratios as separated by Tukey’s HSD (P<0.05).

As leaf number was the only growth measurement collected all three runs and leaf number was highly correlated with rosette diameter in runs two and three (r^2^ = 0.86, *p*<0.001), only leaf number data is presented here. Leaf number was significantly affected by population and treatment, but there were no population by treatment interactions (Table 3). Leaf number was significantly higher in the 0.1∶1 Ca:Mg treatment than in the 1∶0.1 Ca:Mg treatment. Leaf number in the 1∶1 Ca:Mg treatment was intermediate between the other two, but not significantly different from either (Figure 2a). Jug Bay had the highest growth rate, but there was no difference in growth rate between Dover and Pilot (Fig. 2a).

**Table 3:** ANOVA table for rosette growth (leaves per week), biomass, relative water content, and total chlorophyll (*a*+*b*) of three *A. l.* ssp. *lyrata* populations exposed to different substrate Ca to Mg ratios.

	Growth			Biomass			Relative Water Content			Chlorophyll *a*+ *b*		
*Source of Variation*	*df*	F	*P*	*df*	F	*P*	*df*	F	*P*	*df*	F	*P*
Initial Leaf #	1	17.78	<0.0001	1	31.59	<0.0001	1	1.71	0.1949	1	0.03	0.8683
Run	2	5.93	0.0033	2	0.24	0.7879	2	20.80	<0.0001	2	15.11	<0.0001
Block	7	0.59	0.7612	7	0.70	0.6680	3	1.55	0.2088	3	0.78	0.5093
Population	2	12.33	**<0.0001**	2	10.03	**<0.0001**	2	0.01	0.9880	2	0.05	0.9554
Ca to Mg Ratio	2	3.30	**0.0394**	2	0.24	0.1077	2	1.24	0.2933	2	0.79	0.4573
Population x Ca to Mg Ratio	4	0.73	0.5705	4	0.68	0.6059	4	1.04	0.3931	4	1.55	0.1984
Run x Ca to Mg Ratio	4	6.27	0.0001	4	2.00	0.0987	4	0.80	0.5268	4	0.63	0.6417
Error	154			130			66			75		

Above ground biomass was evaluated by measuring both fresh weight and dry weight. Root biomass was not measured, as it was too difficult to extract in its entirety from the rockwool substrate. Fresh weight and dry weight were highly correlated (r^2^ = 0.98, *p*<0.0001), so that only the dry weight data is presented. Only population had a significant effect on dry weight production (Table 3). The Jug Bay (alluvial sandy loam) population consistently had the greatest biomass, while the Dover (limestone sand) and Pilot (serpentine) populations were not significantly different from each other and were lower than Jug Bay (Fig. 2b).

Relative water content did not differ among substrate Ca to Mg ratio or populations (Table 3), with an average RWC of around 79.27±0.27%. Although after visual inspection it appeared that there might be differences in total chlorophyll content among populations there were no significant differences among populations or Ca to Mg ratio with an average chlorophyll content of 7.22±0.53 µmol·g fw^−1^ (Table 3).

**Figure 3:**
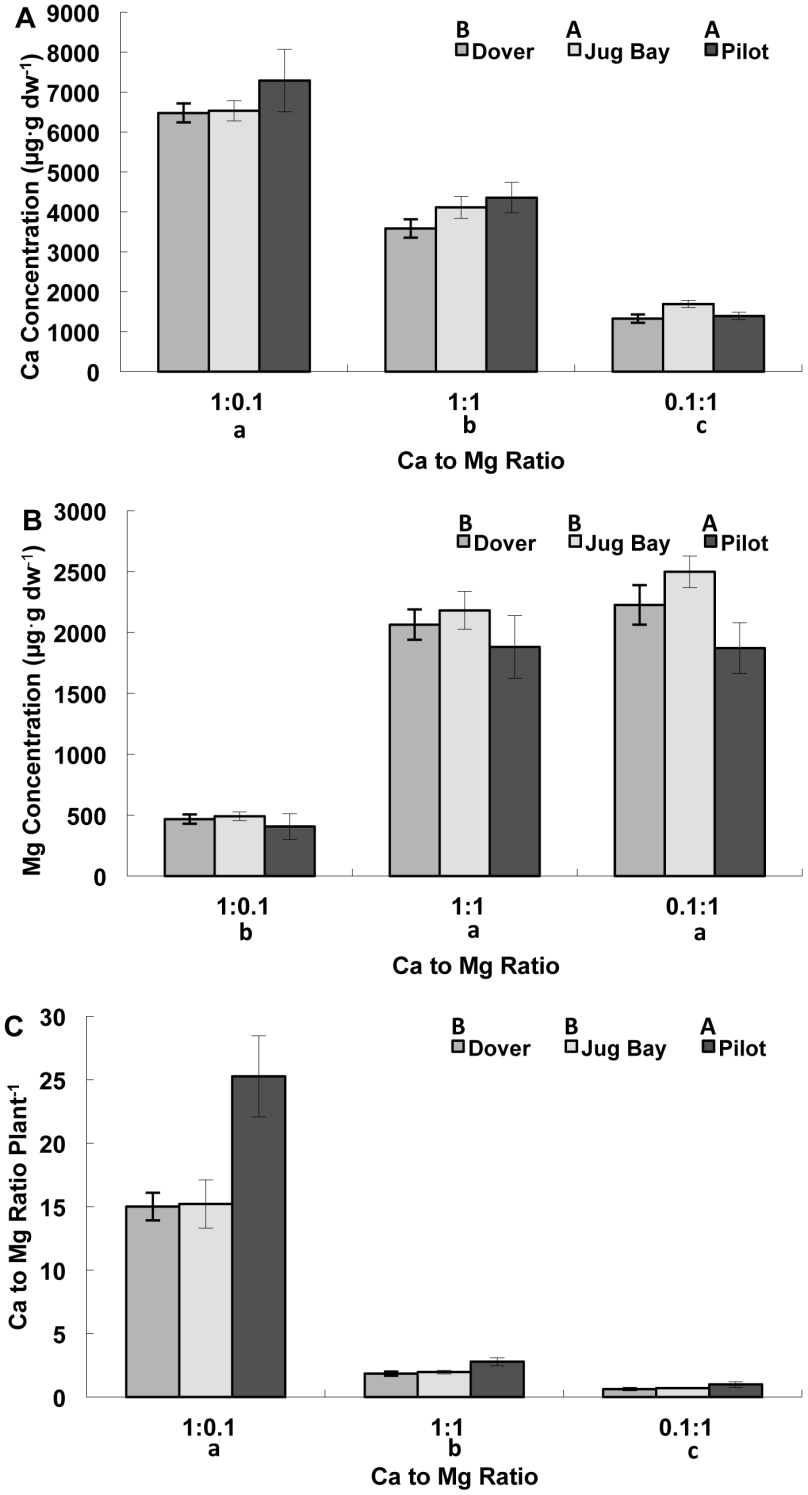
Rosette tissue Ca and Mg concentration of juvenile plants of three *A.l.* ssp. *lyrata* populations. **A.** Rosette tissue Ca concentration**,** **B.** Rosette tissue Mg concentration, and **C.** Rosette tissue Ca to Mg ratio. All data are presented as a mean ± standard error (n = 16). The plants were from serpentine (Pilot), limestone sand (Dover), and sandy loam (Jug Bay) soils. Different upper case letters indicate significant differences among populations, and different lower case letters indicate significant differences among substrate Ca to Mg ratios as separated by Tukey’s HSD (P<0.05).

Calcium and Mg content of the leaves and the leaf Ca to Mg ratio were affected by population and by substrate Ca to Mg ratio, but there were no significant population by treatment interactions (Table 4). The Ca content of the leaf tissue was higher in plants that had more available Ca and lowest in the 0.1∶1 treatment, which had the lowest physical amount of Ca (Fig. 3a). Pilot (serpentine) and Jug Bay (alluvial sandy loam) had significantly higher leaf Ca content than Dover (limestone sand) (Fig. 3a). There was a significant run by treatment effect (Table 4), with the significant effect of population seen mostly in the data from the second run. The trend for leaf Mg content was similar to that seen with leaf tissue Ca, where more Mg was in the leaves of plants grown with more Mg and less Mg in plants grown with less Mg (Fig. 3b), with Pilot having significantly less Mg than Dover and Jug Bay (Fig. 3b). However, unlike in leaf Ca content, where leaf Ca content in the 1∶1 Ca:Mg treatment was intermediate between the other two treatments, leaf Mg content in 1∶1 was not significantly different from the 0.1∶1 Ca:Mg treatment. The significant run by treatment interaction (Table 4) is because Pilot was not significantly different from Dover in run one. Leaf Ca to Mg ratio was also affected by population and treatment, but with no interaction (Table 4). Pilot had significantly higher leaf tissue Ca to Mg ratios than Dover and Jug Bay all three runs (Fig. 3c). The highest leaf tissue Ca to Mg ratio was in the 1∶0.1 treatment while the lowest was in the 0.1∶1 treatment (Fig. 3c), but in all cases the leaf Ca to Mg ratio in Pilot was 1.4–1.7 times greater than in either Dover or Jug Bay.

**Table 4:** ANOVA table for Ca, Mg, and Ca to Mg ratio in the rosettes of three *A. l.* ssp. *lyrata* populations exposed to different substrate Ca to Mg ratios.

	Ca concentration			Mg Concentration			Ca to Mg Ratio		
*Source of Variation*	*df*	F	*P*	*df*	F	*P*	*df*	F	*P*
Initial Leaf #	1	0.04	0.8377	1	0.07	0.7961	1	0.20	0.6571
Run	2	0.28	0.7583	2	5.59	0.0047	2	5.91	0.0035
Block	7	1.00	0.4369	7	1.62	0.1352	7	2.61	0.0148
Population	2	4.55	**0.0123**	2	4.51	**0.0128**	2	11.72	**<0.0001**
Ca to Mg Ratio	2	311.56	**<0.0001**	2	200.74	**<0.0001**	2	775.93	**<0.0001**
Population x Ca to Mg Ratio	4	1.18	0.3218	4	0.12	0.9737	4	0.50	0.7362
Run x Ca to Mg Ratio	4	3.36	0.0119	4	2.76	0.0304	4	7.66	<0.0001
Error	129			129			129		

**Figure 4:**
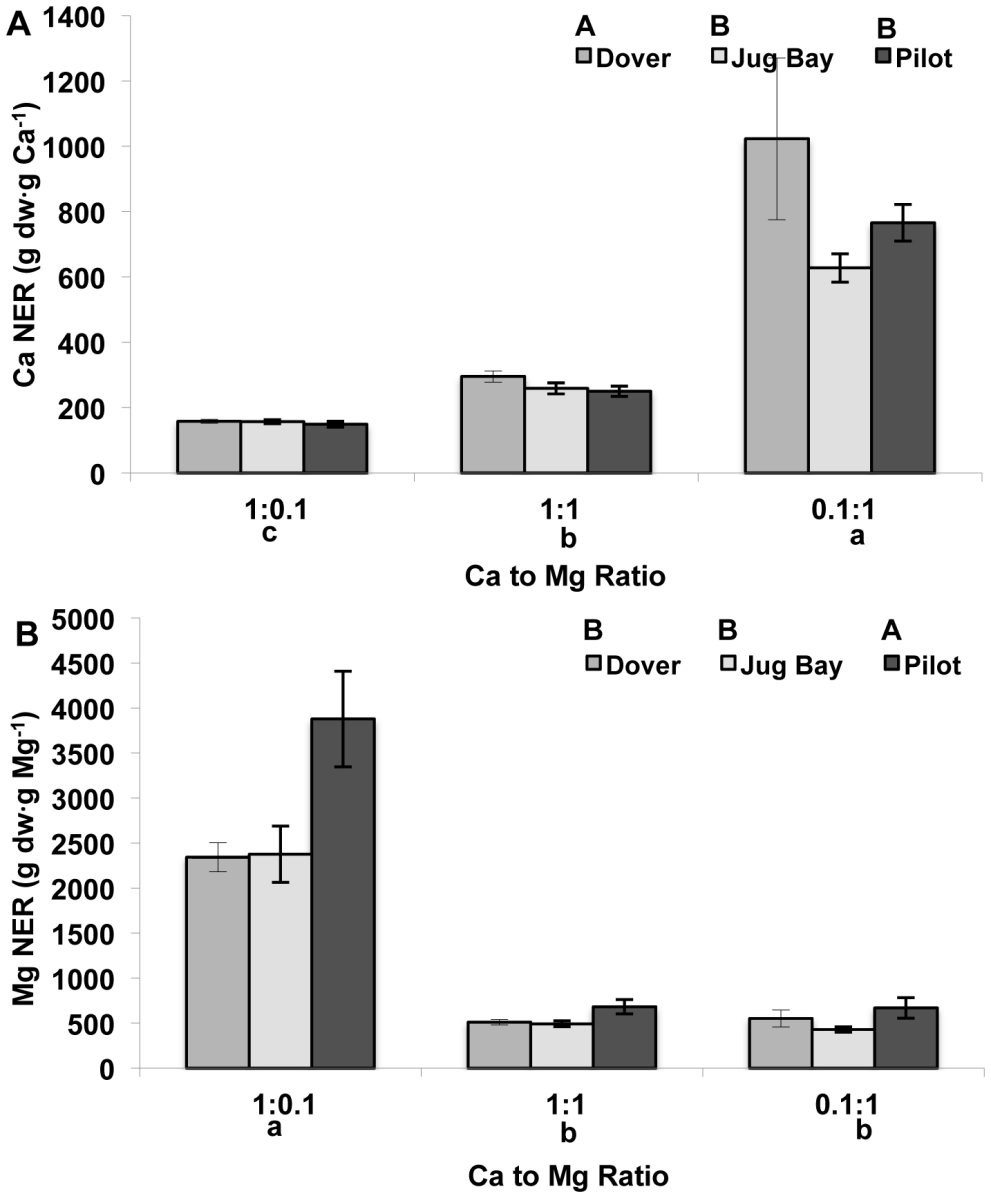
Rosette nutrient efficiency ratio (NER) of juvenile plants of three *A.l.* ssp. *lyrata* populations. **A.** Rosette Ca NER, and **B.** Rosette Mg NER. All data are presented as a mean ± standard error (n = 16). The plants were from serpentine (Pilot), limestone sand (Dover), and sandy loam (Jug Bay) soils. Different upper case letters indicate significant differences among populations, and different lower case letters indicate significant differences among substrate Ca to Mg ratios as separated by Tukey’s HSD (P<0.05).

Calcium NER was significantly affected by both population and by substrate Ca to Mg ratio (Table 5). Calcium NER was significantly different among all three treatments, with the highest NER in the 0.1∶1 treatment and lowest in the 1∶0.1 treatment (Fig. 4a). Dover had significantly higher Ca NER than Pilot and Jug Bay (Fig. 4a). Magnesium NER was also significantly affected by population and by substrate Ca to Mg ratio (Table 5). Magnesium NER was significantly higher in the 1∶0.1 treatment than the other two treatments, and was significantly higher in Pilot than in Dover and Jug Bay (Fig. 4b).

**Table 5:** ANOVA table for Ca and Mg nutrient efficiency ratio (NER) of three *A. l.* ssp. *lyrata* populations exposed to different substrate Ca to Mg ratios.

	Ca NER			Mg NER		
*Source of Variation*	*df*	F	*P*	*df*	F	*P*
Initial Leaf #	1	0.04	0.8377	1	0.07	0.7961
Run	2	0.28	0.7583	2	5.59	0.0047
Block	7	1.00	0.4369	7	1.62	0.1352
Population	2	4.55	**0.0123**	2	4.51	**0.0128**
Ca to Mg Ratio	2	311.56	**<0.0001**	2	200.74	**<0.0001**
Population x Ca to Mg Ratio	4	1.18	0.3218	4	0.12	0.9737
Run x Ca to Mg Ratio	4	3.36	0.0119	4	2.76	0.0304
Error	129			129		

## Discussion

Species with populations in diverse habitats at broad geographical scales either display wide niche breadth due to phenotypically plastic traits, or local adaptation to particular combinations of environmental factors [58]. The bodenvag species, *A. l.* ssp. *lyrata*, exhibits cross-tolerance to the three different low-competition habitats in this study. This plasticity may enable *A. l.* ssp. *lyrata* to successfully colonize new habits, and could explain its highly disjunct distribution. Serpentine soils are known to be particularly stressful, due to low Ca to Mg ratios, other nutrient deficiencies, the presence of heavy metals, and drought [11], [59]. This combination of stressors results in a high level of endemism, and for species that are not endemic they may develop such a high level of specialization (local adaptation) that they lose the ability to grow competitively in other environmental conditions [41], [59]. A recent survey of microsatellite markers in *A. l.* ssp. *lyrata* revealed genetic differentiation between the Dover (limestone sand) and Pilot (serpentine) populations [41], while microarray analysis showed genetic differentiation between populations from granitic and serpentine soils [42], [60]. The three populations in this study are from areas with very different Ca to Mg ratios within the soil (Table 1). With such a diverse array of substrates, and evidence of genetic differentiation, we expected different *A. l.* ssp. *lyrata* populations to show evidence of local adaptation in response to selection to the different Ca to Mg ratios within each substrate and to exhibit this differentiation as home-site advantage in the measured growth parameters [11], [61]–[65].

Although there are three different serpentine populations of *A. l.* ssp. *lyrata* in the Mid-Atlantic region, there are not any comparable populations to pair with the Dover and Jug Bay populations, and even the serpentine populations vary widely in soil parameters (unpublished data). In our studies, the hallmark of home site advantage would be seen as treatment by population interactions in germination success, germination rate, root growth rate in seedlings, and treatment by population interactions in leaf number and biomass in rosettes. However, the populations of *A. lyrata* ssp. *lyrata* studied here do not show signs of home site advantage as evidenced by physiological differentiation to soil Ca:Mg conditions, as there were no significant treatment by population interactions in any of those measurements. Instead, the populations of *A. lyrata* ssp. *lyrata* studied here exhibit varying degrees of cross tolerance to a range of Ca:Mg ratios. Two main nutrient factors make serpentine soils difficult to live in: low Ca:Mg ratios and the presence of heavy metals such as Ni and Cr [20]–[22], [35], [39]–[40]. As there were such differences among the soils of each population we decided to look at the main nutrient differences separately rather than dealing with all of the interacting factors that are inherent in reciprocal transplants into home soil. However, we would not be surprised if these populations show a differential response to heavy metals.

Differences in population growth responses to substrate Ca to Mg ratio were not evident in early life stages, including germination and radicle growth. The serpentine population (Pilot) had the lowest overall germination rate (Fig. 1 b); both the serpentine and calcareous (Dover) populations had the lowest overall radicle growth rate (Fig. 1c); however, these reductions were found in all treatments. This low germination rate has previously been observed in Pilot and one other serpentine population of *A. l.* spp. *lyrata* [66], and has also been observed in serpentine versus non-serpentine ecotypes of weedy and non-weedy species in Pennsylvania [67]. However, in other species there is no difference in germination rate between serpentine and non-serpentine ecotypes [68]–[69]. During the juvenile rosette stage, before the onset of flowering, there were also growth differences among populations, but the magnitude of the differences did not vary by substrate. Plants from the Jug Bay (alluvial sandy loam) population consistently had the highest growth rates and biomass production, while the Dover (calcareous sand) and Pilot (serpentine) (Figs. 2 and 3) populations were not significantly different from each other. The three habitats that the plants are from are quite different from one another in other dimensions besides Ca:Mg ratios: Pilot and Dover both have very scant humus in the substrates while Jug Bay has a richer humus factor. This could change the water availability to plants, especially during dry periods, and Pilot and Dover populations may divert resources to drought resistance traits, thus limiting their growth rates even under conditions of ample water availability (as in this experiment). Under ambient conditions in the lab, Jug Bay substrate had the highest water holding capacity after 24 hours (32%), followed by Dover substrate (24%), and then Pilot substrate (1.5%) (unpublished data). The NPK profile of substrates was also different among the three: all had high K, but nitrate was lowest in Dover, medium in Pilot, and highest in Jug Bay, and P was very low in Pilot, compared to low in Jug Bay and Dover (unpublished data).

The only evidence of special adaptation (i.e. physiological differentiation) in relation to substrate Ca to Mg ratio we observed was in the rosette Ca to Mg ratio (Fig. 3c). Plants from Pilot (serpentine) were able to maintain a higher internal Ca to Mg ratio than the plants from Jug Bay (alluvial sandy loam) and Dover (calcareous sand) regardless of whether Ca or Mg was abundant or scarce. This could be due to a highly efficient Ca uptake mechanism or to a highly effective Mg exclusion mechanism. Currently there is little evidence for any Mg exclusion mechanisms in *Arabidopsis* species; however, a recent study by Turner *et al.* [60] revealed genetic differentiation in genes coding for Mg transport between granitic and serpentine populations of *A. l.* ssp. *lyrata.* There is more evidence for differences in Ca transport in *Arabidopsis* species, such as a study by Bradshaw [40] that found that *A. thaliana* with a mutation in *cax1* (a Ca^2+^/H^+^ antiporter) exhibited greater tolerance to low Ca to Mg ratios, such as those found in serpentine soils, than the wild type plants. The *cax1* mutants also exhibited other traits that would be adaptive in a serpentine environment, such as low leaf Mg concentrations. Turner *et al.* [42], [60] found differentiation among serpentine and granitic populations of *A. l.* ssp. *lyrata* for genes related to Ca homeostasis, some of which may contribute to the higher internal Ca to Mg ratio seen in the Pilot serpentine population (Fig. 3c). There are particularly low levels of diversity at these loci in the serpentine populations, which is consistent with strong purifying selection against gene flow at these loci with important function in Ca ion transport in the extreme serpentine environment [42], [60]. Maintaining as high of an internal Ca to Mg ratio as possible is favorable to plant function [2]–[3] and could be particularly advantageous in serpentine soils where Ca concentrations are limiting and Mg concentration may be toxic [35], [39]–[40]. Since seeds were field collected, maternal effects could also have played a role in the differences among populations that we observed, particularly the differences in seed germination and early seedling growth [70]. However, due to the similarity in other growth parameters among populations the maternal effects on these parameters appear to be minimal, which is consistent with studies that show that environmental maternal effects decline as the plant grows [69]–[70]. In these three populations there did not appear to be any signs of Mg toxicity in the serpentine like conditions; therefore, the relatively high internal Ca to Mg ratio within the serpentine population may have a different function, such as helping serpentine populations deal with heavy metal stress [71]–[73].

The Ca concentration in the 0.1∶1 treatment (16 µg/g) is very close to that extractable Ca in the serpentine soils found at the Pilot location (13 µg/g), while the Mg concentration was more than twice as high (96 µg/g) as the extractable Mg in Pilot soils (39 µg/g). All of the plants grew very well under these serpentine like conditions (as measured by growth rate and biomass production), and the serpentine population did not have reduced growth under substrates where Ca concentration was as high as or higher than the Mg concentration, which might be expected in populations that have experienced strong selection under the opposite conditions [11], [38], [62], [74]. The two populations that come from environments with extreme Ca to Mg ratio (calcareous sand and serpentine) did not differ significantly in biomass production, while the Jug Bay population had highest growth under all treatment conditions (Fig. 2). Also of interest, Dover had significantly greater Ca NER than Pilot and Jug Bay, and Pilot had significantly greater Mg NER than Jug Bay and Dover (Fig. 4). At least one other serpentine species (*Agropyron spicatum*) has demonstrated the ability to maintain consistent production under both low and high Mg concentrations [75]. However, all three *A. l.* ssp. *lyrata* populations in our study were able to maintain a high NER under conditions where Ca or Mg were deficient (Fig. 4), which is not uncommon in plants that are from nutrient deficient habitats [76]–[78]. As the soil from Jug Bay is much more acidic than those from Dover and Pilot (Table 1) it may naturally encounter deficiencies in available Ca and Mg [57], which may account for the ability of the Jug Bay population to grow well under all three Ca to Mg ratios. In addition the soil from Jug Bay had lower levels of total Ca and Mg than both soils from Dover and Pilot, with the lowest extractable Mg of the three soil types (Table 1).

These data indicate that there is cross-tolerance in these three populations to both low and high Ca and Mg concentrations. This could be effective in dealing with all ion excess/deficit or may specifically apply to only these two divalent cations. Populations that grow under harsh conditions are often able to grow under other seemingly unrelated stressful conditions [11], [59]. Tolerance to a wide range of Ca to Mg ratios may be a constitutive trait in *A. l.* ssp. *lyrata*, or may be specific to these three populations. This type of cross-tolerance has also been observed in constitutive metal tolerance between serpentine and calcareous populations of *Thlaspi goesingense* [59] and has been observed for Zinc tolerance among diverse populations of *A. halleri* [45], [64], [79]. Cross-tolerance cannot be explained by cross-pollination [59] occurring among our *A. l.* ssp. *lyrata* populations, as they are all at least 100 km from each other with few isolated intervening populations. Although the internal Ca to Mg ratio supports the hypothesis of physiological differentiation through the juvenile stage, all of the other data that was collected supports the hypothesis that adaptation to different substrate Ca to Mg ratios is due to a more generalist strategy within *A. l.* ssp. *lyrata*, resulting in cross-tolerance.

## References

[1] Main JL (1981) Magnesium and calcium nutrition of a serpentine endemic grass. Am Midl Nat 105: 196–199.

[2] O’Dell RE, James JJ, Richards JH (2006) Congeneric serpentine and nonserpentine shrubs differ more in leaf Ca:Mg than in tolerance of low N, low P, or heavy metals. Plant Soil 280: 49–64.

[3] Asemaneh T, Ghaderian SM, Baker AJM (2007) Responses to mg/ca balance in an Iranian serpentine endemic plant, *Cleome heratensis* (Capparaceae) and a related non-serpentine species, *C. foliolosa* . Plant Soil 293: 49–59.

[4] Anacker BL, Whittall JB, Goldberg EE, Harrison SP (2010) Origins and consequences of serpentine endemism in the California flora. Evolution 65: 365–376. 20812977 10.1111/j.1558-5646.2010.01114.x

[5] Pope N, Harris TB, Rajakaruna N (2010) Vascular plants of adjacent serpentine and granite outcrops on the deer isles, Maine, U.S.A. Rhodora. 112: 105–141.

[6] Woodell SRJ, Mooney HA, Lewis H (1975) The adaptation to serpentine soils in California of the annual species *Linanthus androsaceus* (Polemoniaceae). B Torrey Bot Club 102: 232–238.

[7] Marrs RH, Proctor J (1976) The response of serpentine and non-serpentine *Agrostis stolonifera* to magnesium and calcium. J Ecol 64: 953–964.

[8] Johnston WR, Proctor J (1981) Growth of serpentine and non-serpentine races of *Festuca rubra* in solutions simulating the chemical conditions in a toxic serpentine soil. J Ecol 69: 855–869.

[9] Rajakaruna N, Yaeesh Siddiqi M, Whitton J, Bohm BA, Glass ADM (2003) Differential responses to Na^+^/K^+^ and Ca^2+^/Mg^2+^ in two edaphic races of the *Lasthenia californica* (Asteraceae) complex: A case for parallel evolution of physiological traits. New Phytol 157: 93–103. 10.1046/j.1469-8137.2003.00648.x 33873697

[10] Lortie CJ, Aarssen LW (1996) The specialization hypothesis for phenotypic in plants. Int J Plant Sci 157: 484–487.

[11] Murren CJ, Douglass L, Gibson A, Dudash MR (2006) Individual and combined effects of Ca/Mg ratio and water on trait expression in *Mimulus guttatus* . Ecology 87: 2591–2602. 17089667 10.1890/0012-9658(2006)87[2591:iaceom]2.0.co;2

[12] Bradshaw AD (1965) Evolutionary significance of phenotypic plasticity in plants. Advan Genet 13: 115–155.

[13] Schlichting CD (1986) The evolution of phenotypic plasticity in plants. Annu Rev Ecol Syst 17: 667–693.

[14] Pigliucci M (2005) Phenotypic plasticity: where are we going now? Trends Ecol Evol 20: 481–486. 16701424 10.1016/j.tree.2005.06.001

[15] Cooper A (1976) The vegetation of carboniferous limestone soils in South Wales: II. Ecotypic adaptation in response to calcium and magnesium. J Ecol 64: 147–155.

[16] Going BM, Hillerislambers J, Levine JM (2009) Abiotic and biotic resistance to grass invasion in serpentine annual plant communities. Oecologia 159: 839–847. 19139921 10.1007/s00442-008-1264-y

[17] McGahan DG, Southard RJ, Claassen VP (2009) Plant-available calcium varies widely in soils on serpentinite landscapes. Soil Sci Soc Am J 73: 2087–2095.

[18] Cooper A, Etherington JR (1974) The vegetation of carboniferous limestone soils in south Wales: I. dolomitization, soil magnesium status and plant growth. J Ecol 62: 179–190.

[19] Kataeva MN, Alexeeva-Popova NT, Drozdova IV, Beljaeva AI (2004) Chemical composition of soils and plant species in the Polar Urals as influenced by rock type. Geoderma 122: 257–268.

[20] Briscoe LRE, Harris TB, Broussard W, Dannenberg E, Olday FC, et al (2009) Bryophytes of adjacent serpentine and granite outcrops on the deer isles, Main, U.S.A. Rhodora. 111: 1–20.

[21] Shaw AJ (1991) Ecological genetics of serpentine tolerance in the moss, *Funaria flavicans*: variation within and among haploid sib families. Am J Bot 78: 1487–1493.

[22] Ghasemi R, Ghaderian SM (2009) Responses of two populations of an Iranian nickel-hyperaccumulating serpentine plant, *Alyssum inflatum* Nyar., to substrate Ca/Mg quotient and nickel. Environ Exp Bot 67: 260–268.

[23] Johnston WR, Proctor J (1984) The effects of magnesium, nickel, calcium and micronutrients on the root surface phosphatase activity of a serpentine and non-serpentine clone of *Festuca rubra* L. New Phytol. 96: 95–101.

[24] Palm E, Brady K, Van Volkenburgh EV (2012) Serpentine tolerance in *Mimulus guttatus* does not rely on exclusion of magnesium. Funct Plant Biol 39: 679–688. 10.1071/FP12059 32480819

[25] Shewry PR, Peterson PJ (1975) Calcium and magnesium in plants and soil from a serpentine area on Unst, Shetland. J Appl Ecol 12: 381–391.

[26] Lyon GL, Peterson PJ, Brooks RR, Butler GW (1971) Calcium, magnesium and trace elements in a New Zealand serpentine flora. J Ecol 59: 421–429.

[27] O’Kane SL, Al-Shehbaz IA (1997) A synopsis of *Arabidopsis* (Brassicaceae). Novon 7: 323–327.

[28] Mitchell-Olds T (2001) *Arabidopsis thaliana* and its wild relatives: a model system for ecology and evolution. TRENDS Ecol Evol 16: 693–700.

[29] Riihimaki M, Savolainen O (2004) Environmental and genetic effects on flowering differences between northern and southern populations of *Arabidopsis lyrata* (Brassicaceae). Am J Bot 91: 1036–1045. 21653459 10.3732/ajb.91.7.1036

[30] Mable BK, Robertson AV, Dart S, Di Berardo C, Witham L (2005) Breakdown of self-incompatibility in the perennial *Arabidopsis lyrata* (Brassicaceae) and its genetic consequences. Evolution 59: 1437–1448. 16153030

[31] Clauss MJ, Dietel S, Schubert G, Mitchell-Olds T (2006) Glucosinolate and trichome defenses in a natural *Arabidopsis lyrata* population. J Chem Ecol 32: 2351–2373. 17089185 10.1007/s10886-006-9150-8

[32] Mable BK, Adam A (2007) Patterns of genetic diversity in outcrossing and selfing populations of *Arabidopsis lyrata* . Mol Ecol 16: 3565–3580. 17845431 10.1111/j.1365-294X.2007.03416.x

[33] Hart R (1980) The coexistence of weeds and restricted native plants on serpentine barrens in southeastern Pennsylvania. Ecology 61: 688–701.

[34] Walck JL, Baskin JM, Baskin CC (1999) Relative competitive abilities and growth characteristics of a narrowly endemic and a geographically widespread *Solidago* species (Asteraceae). Am J Bot 86: 820–828. 10371724

[35] Proctor J (1999) Toxins, nutrient shortages and droughts: The serpentine challenge. TREE 14: 334–335.

[36] Løe G, Toräng P, Gaudeul M, Årgen J (2007) Trichome production and spatiotemporal variation in herbivory in the perennial herb *Arabidopsis lyrata* . Oikos 116: 134–142.

[37] Kooijman AM, Jongejans J, Sevink J (2005) Parent material effects Mediterranean woodland ecosystems in NE Spain. Catena 59: 55–68.

[38] Powell KI, Knight TM (2009) Effects of nutrient addition and competition on biomass of five *Cirsium* species (Asteraceae), including a serpentine endemic. Int J Plant Sci 170: 918–925.

[39] Spence DHN, Millar EA (1963) An experimental study of the infertility of a Shetland serpentine soil. J Ecol 51: 333–343.

[40] Bradshaw HD Jr (2005) Mutations in CAX1 produce phenotypes characteristic of plants tolerant to serpentine soils. New Phytol 167: 81–88. 15948832 10.1111/j.1469-8137.2005.01408.x

[41] Lloyd MW, Roche B, Roberts R (2011) Genetic variation and population structure of *Arabidopsis lyrata* spp. *lyrata* (Brassicaceae) along the eastern seaboard of North America. Castanea 76: 28–42.

[42] Turner TL, von Wettberg EJ, Nuzhdin SV (2008) Genomic analysis of differentiation between soil types reveals candidate genes for local adaptation in *Arabidopsis lyrata* . Plos One 3: e3183 doi:10.1371/journal.pone.0003183. 18784841 10.1371/journal.pone.0003183 PMC2527522

[43] Grusak MA (1994) Iron transport to developing ovules of *Pisum sativum*. 1. Seed import characteristics and phloem iron-loading capacity of source regions. Plant Physiol 104: 649–655. 12232115 10.1104/pp.104.2.649 PMC159243

[44] Pomper KW, Grusak MA (2004) Calcium uptake and whole-plant water use influence pod calcium concentration in snap bean plants. J Am Soc Hortic Sci 129: 890–895.

[45] Bert V, Macnair MR, De Laguerie P, Saumitou-Laprade P, Petit D (2000) Zinc tolerance and accumulation in metallicolous and nonmetallicolous populations of *Arabidopsis halleri* (Brassicaceae). New Phytol 146: 225–233. 10.1046/j.1469-8137.2000.00634.x 33862970

[46] Mustart PJ, Cowling RM (1993) The role of regenerate stages in the distribution of edaphically restricted *Fynbos proteaceae* . Ecology 74: 1490–1499.

[47] Harris RC (2009) Four novel lichen taxa in the lichen biota of eastern North America. Opuscula Philolichenum 6: 149–156.

[48] Khan H, Brush GS (1994) Nutrient and metal accumulation in a freshwater tidal marsh. Estuaries 17: 345–360.

[49] Maguire JD (1962) Speed of germination aid in selection and evaluation for seedling emergence and crop vigor. Crop Sci 2: 176–177.

[50] Rasband WS (1997–2005) ImageJ, U. S. National Institutes of Health, Bethesda, Maryland, USA, http://rsb.info.nih.gov/ij/.

[51] JMP Version 8 (1989–2008) SAS Institute Inc., Cary, NC.

[52] Epstein E, Bloom AJ (2005) Mineral nutrition of plants: Principles and perspectives, 2^nd^ edition. Sinauer Associates, Sunderland, MA, p.31.

[53] Bogeat-Triboulot MB, Brosche M, Renaut J, Jouve L, Le Thiec D, et al (2007) Gradual soil water depletion results in reversible changes of gene expression, protein profiles, ecophysiology, and growth performance in *Populus euphratica*, a poplar growing in arid regions. Plant Physiol 143: 876–892. 17158588 10.1104/pp.106.088708 PMC1803728

[54] Inskeep WP, Bloom PR (1985) Extinction coefficients of chlorophyll a and b in N,N-dimethylformamide and 80% acetone. Plant Physiol 77: 483–485. 16664080 10.1104/pp.77.2.483 PMC1064541

[55] Reiss C (1994) Experiments in Plant Physiology. Prentice-Hall, Upper Saddle River, NJ, 24–25.

[56] Olsen KG, Ulicny LJ (2001) Reduction of calcium concentrations by the Brita water filtration system: a practical experiment in titrimetry and atomic absorption spectroscopy. J Chem Educ 78: 941.

[57] Baligar VC, Fageria NK, He ZL (2001) Nutrient use efficiency in plants. Commun Soil Sci Plan 32: 921–950.

[58] Bennington CC, Fletcher N, Vavrek MC, Shaver GR, Cummings KJ, et al (2012) Home site advantage in two long-lived arctic plant species: results from two 30-year reciprocal transplant studies. J Ecol 100: 841–851.

[59] Brady KU, Kruckeberg AR, Bradshaw HD Jr (2005) Evolutionary ecology of plant adaptation to serpentine soils. Annu Rev Ecol Syst 36: 243–266.

[60] Turner TL, Bourne EC, von Wettberg EJ, Hu TT, Nuzhdin SV (2010) Population resequencing reveals local adaptation of *Arabidopsis lyrata* to serpentine soils. Nat Gen 42: 260–264. 10.1038/ng.515 20101244

[61] Silander JA Jr (1985) The genetic basis of the ecological amplitude of *Spartinia patens*. II. Variance and correlation analysis. Evolution 39: 1034–1052. 28561514 10.1111/j.1558-5646.1985.tb00445.x

[62] Baythavong BS (2011) Linking the spatial scale of environmental variation and the evolution of phenotypic plasticity: Selection favors adaptive plasticity in fine-grained environments. Am Nat 78: 000–000 Doi:10.1086/660281. 10.1086/660281 21670579

[63] Galloway LF, Fenster CB (2000) Population differentiation in an annual legume: local adaptation. Evolution 54: 1173–1181. 11005286 10.1111/j.0014-3820.2000.tb00552.x

[64] Kingsolver JG, Pfennig DW, Servidio MR (2002) Migration, local adaptation and the evolution of plasticity. Trends Ecol Evol 17: 540–541.

[65] Schlötterer C (2002) Towards a molecular characterization of adaptation in local populations. Curr Opin Genet Dev 12: 683–687. 12433582 10.1016/s0959-437x(02)00349-0

[66] Veatch-Blohm ME, Koutavas E (2011) The effect of stratification and after-ripening time on seed germination of three populations of *Arabidopsis lyrata* ssp. *lyrata* (Brassicaceae). Castanea 76: 199–209.

[67] Hart R (1980) The coexistence of weeds and restricted native plants on serpentine barrens in southeastern Pennsylvania. Ecology 61: 688–701.

[68] Reeves RD, Baker AJM (1984) Studies on metal uptake by plants from serpentine and non-serpentine populations of *Thlaspi goesingense* Halacsy (Cruciferae). New Phytol 98: 191–204. 10.1111/j.1469-8137.1984.tb06108.x 29681122

[69] Wright JW, Stanton ML (2007) *Collinsia sparsiflora* in serpentine and nonserpentine habitats: Using F2 hybrids to detect the potential role of selection in ecotypic differentiation. New Phytol 173: 354–366. 17204082 10.1111/j.1469-8137.2006.01925.x

[70] Bischoff A, Müller-Schärer H (2010) Testing population differentiation in plant species – how important are environmental maternal effects. Oikos 199: 445–454.

[71] Lombini A, Llugany M, Poschenrieder C, Dinelli E, Barcelo J (2003) Influence of Ca/Mg ratio on Cu resistance in three *Silene armeria* ecotypes adapted to calcareous soil or to different, Ni- or Cu-enriched. Serpentine sites. J Plant Physiol 160: 1451–1456. 14717437 10.1078/0176-1617-01002

[72] Chaney RL, Chen KY, Li YM, Angle JS, Baker AJM (2009) Effects of calcium on nickel tolerance and accumulation in *Alyssum* species and cabbage grown in nutrient solution. Plant Soil 311: 131–140.

[73] Mieczek M, Kozlowska M, Kaczmarek Z, Chadzinikolau T, Golinski P (2012) Influence of Ca/Mg ratio on phytoextraction properties of *Salix viminalis* I. The effectiveness of Cd, Cu, Pb, and Zn bioaccumulation and plant growth. Int J Phytoremediat 14: 75–88. 10.1080/15226514.2011.573824 22567696

[74] Harrison S, Cornell H, Moore KA (2010) Spatial niches and coexistence: testing theory with tarweeds. Ecology 91: 2141–2150. 20715636 10.1890/09-0742.1

[75] Main JL (1984) Differential responses to magnesium and calcium by native populations of *Agropyron spicatum* . Am J Bot 61: 931–937.

[76] Enoki T, Kawaguchi H, Iwatsubo G (1997) Nutrient-uptake and nutrient-use efficiency of *Pinus thunbergii* Parl. Along a topographical gradient of soil nutrient availability. Ecol Res 12: 191–199.

[77] Sing SP, Bisht K (1992) Nutrient utilization in *Quercus leucotrichophora* and *Pinus roxburghii* seedlings at five soil fertility levels. J Veg Sci 3: 573–578.

[78] Sultan SE, Bazzaz FA (1993) Phenotypic plasticity in *Polygonum persicaria*. III. The evolution of ecological breadth for nutrient environment. Evolution 47: 1050–1071. 28564292 10.1111/j.1558-5646.1993.tb02134.x

[79] Marques L, Cossegal M, Bodin S, Czernic P, Lebrun M (2004) Heavy metal specificity of cellular tolerance of two hyperaccumulating plants, *Arabidopsis halleri* and *Thlaspi caerulescens* . New Phytol 164: 289–295. 10.1111/j.1469-8137.2004.01178.x 33873551

